# MicroRNA-22-3p targeted regulating transcription factor 7-like 2 (TCF7L2) constrains the Wnt/β-catenin pathway and malignant behavior in osteosarcoma

**DOI:** 10.1080/21655979.2021.2003942

**Published:** 2022-05-03

**Authors:** YuanLiang Xue, Ya Guo, Ning Liu, XiangQi Meng

**Affiliations:** a Department of Orthopedics, Clinical Medical College of Shandong Uiniversity of Traditional Chinese Medicine, JiNan City, ShanDong Province, China; b Department of Orthopedics, Heze Hospital of Traditional Chinese Medicine, HeZe City, ShanDong Province, China; c Department of Spinal Orthopedics, Zhang Qiu District Hospital of Traditional Chinese Medicine, JiNan City, ShanDong Province, China; d Department of Orthopedics, Suzhou Tcm Hospital Affiliated to Nanjing University of Tcm, SuZhou City, JiangSu Province, China

**Keywords:** MicroRNA-22-3p, transcription factor 7-like 2, osteosarcoma, Wnt/β-catenin, proliferation

## Abstract

Various studies have manifested that microRNAs (miRNAs) are involved in the modulation of the occurrence and development of osteosarcoma (OS). However, whether miR-22-3p is associated with OS growth remains unclear. In the study, the potential molecular mechanisms of miR-22-3p in OS was explored. It was affirmed that miR-22-3p was associated with distant metastasis and tumor size in OS patients, and reduced in OS tissues and cells while transcription factor 7-like 2 (TCF7L2) was elevated. Elevated miR-22-3p repressed OS cell progression, and the Wnt/β-catenin pathway, while elevated TCF7L2 was opposite. MiR-22-3p targeted TCF7L2 in OS. In functional rescue experiments, knockdown of miR-22-3p on OS progression and promotion of Wnt/β-catenin were reversed by simultaneous knockdown of TCF7L2. Transplantation experiments in nude mice showed that elevated miR-22-3p repressed OS tumor growth and decreased TCF7L2, Wnt and β-catenin. Shortly, this study suggest that miR-22-3p refrains the Wnt/β-catenin pathway by targeting TCF7L2 and thereby preventing OS deterioration. MiR-22-3p/TCF7L2 axis is supposed to be a candidate molecular target for future OS treatment.

## Graphical Abstract

**Figure:**
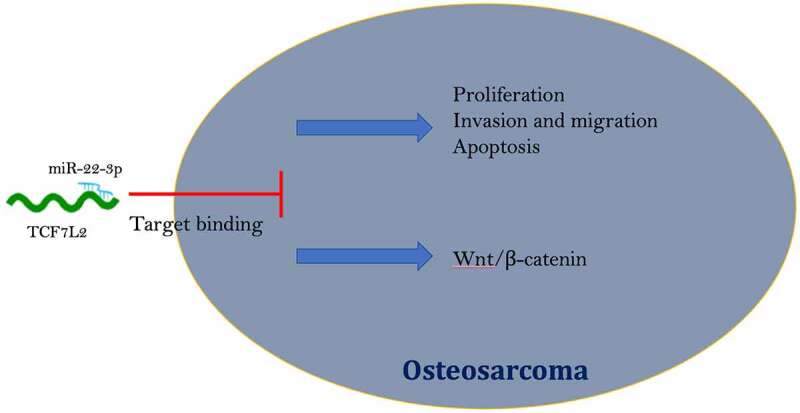

## Highlights

- Descending miR-22-3p but elevated TCF7L2 were in OS.
- MiR-22-3p targeted the expression of TCF7L2 in OS.
- MiR-22-3p repressed OS proliferation, invasion and migration via modulating TCF7L2.
- MiR-22-3p prevented the activation of wnt/β-catenin signaling pathway in OS via controlling TCF7L2.

## 1 Introduction

Osteosarcoma (OS), a bone tumor, performing in adolescents and children, is characterized by rapid progression, high malignancy, poor prognosis, and a tendency to develop lung metastases [1,2]. Although the survival in patients with OS could be prolonged via surgery or radiation therapy, the OS prognosis is still poor, mainly owing to the chemical resistance of OS, especially in patients with metastatic cancer [3–5]. Due to these factors, it is crucial to explore the potential mechanism of OS progression for new therapeutic methods and targets.

MicroRNAs (miRNAs) not encoding genes repress post-transcriptional translation of mRNA by binding to specific sites of target genes and thus influence disease progression [6–8]. Recently, exploring the character of miRNAs in cancer development has become a research hotspot. Recent studies have manifested that both bioengineered miR-34a-5p and −124-3p can suppress tumor growth and spontaneous lung metastasis of raw OS [9]. Wang Y *et al*. reported that miR-557 depresses malignant behavior of OS cells by reducing HOXB9 and inactivating the EMT process [10]. In addition, other studies have suggested that miR-485-3p can suppress c-Met and AKT3/mTOR signaling for knockout of glycolysis and metastasis of OS [11]. As a member of the miRNA family, miR-22-3p has been clarified to be under-expressed in various cancers and to take part in the development of cancers. A recent study reported the suppression of the growth of triple-negative breast cancer via ncRNA treatment with miR-22-3p [12]. Du Y *et al*. found that exogenous up-regulation of miR-22-3p constrains LINC01137 in oral squamous cell carcinoma (OSCC) and OSCC progression [13]. In addition, other studies have informed that miR-22-3p controls GINS2 to suppress the proliferation and strengthen apoptosis of bladder cancer [14]. However, no studies have reported the differential expression and biological function of miR-22-3p in OS.

Wnt, a type of secretory glycoproteins, serves as either autocrine or paracrine [15]. After secretion, Wnt interacts with cell surface specific receptors, inducing β-catenin accumulation [16]. β-catenin, a multifunctional protein, enters the nucleus and regulates gene expression in a free status, in which the abnormality or activation may cause tumors [17,18]. Recently, there are a great many studies reporting that miRNA can be affective in the malignant phenotype of cancer with interaction of the Wnt/β-catenin pathway [19–21]. In the meantime, miR-22-3p has been announced to control Wnt/β-catenin pathway in glioblastoma, whereas the character of miR-22-3p on Wnt/β-catenin pathway in OS is

still ambiguous.

Based on this, the biological function of miR-22-3p/transcription factor 7-like 2 (TCF7L2) axis in OS by silencing or elevating miR-22-3p and TCF7L2, was explored originally in this study. In addition, the effect of miR-22-3p/TCF7L2 axis on the Wnt/β-catenin pathway in OS was examined.

## 2 Methods

### 2.1 Clinical tissue collection

During 2011’2014, Primary OS and para-cancerous tissues were obtained from biopsies of 43 patients diagnosed with primary OS from Xiangya Hospital of Central South University. Prior to surgery, the patient did not receive chemotherapy or radiation therapy. Fresh tissues were frozen and preserved in liquid nitrogen immediately after surgery. Ahead of the study, written informed consent was achieved from each patient, and all study protocols were approved by the Medical School Committee of Xiangya Hospital of Central South University.

### 2.2 Cell culture

Human OS cell lines (MG63, U-2OS, HOS, and SaOS-2) and human normal osteoblast cell lines (hFOB1.19) (the US Typical Culture Preservation Center) were incubated in Dulbecco’s Modified Eagle Medium (DMEM) (Sangon Biotech Co., Ltd.) with 10% fetal bovine serum (FBS) (Gibco; Thermo Fisher Scientific, Inc.), and Roswell Park Memorial Institute (RPMI)-1640 (Hyclone; Cytiva) with 10% FBS separately.

### 2.3 Cell transfection

Small interfering RNA (siRNA) targeting TCF7L2 and si-TCF7L2/NC, TCF7L2 overexpressing plasmid and pcDNA3.1-TCF7L2/NC, and analogue and inhibitor of miR-22-3p and its NC were applied. MG63 and HOS cells (5 × 10^6^ cells/mL) were seeded into a 6-well plate and transfected with the reagents with 70% confluence and Lipofectamine 3000 (Invitrogen) and the manufacturer’s instructions accordingly. Then MG63 and HOS cells were collected for subsequent studies. The above plasmids were synthesized and provided by Shanghai Genomics Pharmaceutical Co., Ltd. The above oligonucleotides were purchased from RioboBio (Guangzhou, China).

### 2.4 Flow cytometry

MG63 and HOS cells were detached with 0.25% trypsin and cell suspension was prepared. After centrifugation at 1200 × g, apoptosis was assessed applying the Annexin V-fluoresceinisothiocyanat (FITC) kit (BD Biosciences) on the grounds of the manufacturer’s protocol. The cells were incubated with a binding buffer including 5 µL Annexin V fluoresceinisothiocyanat (FITC) and 5 µL propidium iodide (PI) in darkness. The cell apoptosis was then detected within 1 h with FACSCalibur flow cytometry (BD Biosciences) and analyzed via CellQuest software (BD Biosciences).

### 2.5 Cell counting kit (CCK)-8 assay

MG63 and HOS cells were seeded (2 × 10^4^ cells/well) into 96-well plates and cultured. After incubation of 0, 24, 48, and 72 h, 10 μL CCK-8 (Biyuntian Biotechnology Institute) was added to the well and cultured for 4 h. The absorbance was measured at 450 nm with a microplate meter. Each well value was read for 3 times.

### 2.6 Cell scratch test

MG63 and HOS cell migration was detected by scratch assay. Shortly, the 6-well plate was marked and horizontal lines were drawn evenly with a ruler, passing through the wells approximately every 0.5’1 cm. At least five lines were through each well. The cells were incubated overnight in labeled petri dishes on the outsoles, and in serum-free medium. The wound area was recorded at 0 h and 24^th^ h by phase contrast microscopy (Olympus MK, Tokyo, Japan).

### 2.7 Transwell

Transwell chamber (8 µm well; BD Biosciences) was adopted to identify cell invasion [22]. In brief, 5 × 10^4^MG63 and HOS cells were seeded in a matrigel-coated upper incubator in serum-free RPMI-1640 medium. Meanwhile, RPMI-1640 medium (500 µL) with 10% FBS was spread in the lower chamber. Then the invasive cells in the lower chamber were fixed with 100% methanol and left with 0.1% stained crystal violet. The stained cells were counted via a light microscope in five randomly selected areas.

### 2.8 Real-time quantitative fluorescence PCR (RT-qPCR)

RT-qPCR was performed as previously described [23]. The RNA from cells and tissues was extracted with Trizol Extract® Reagents (Invitrogen; Thermo Fisher Scientific, Inc.). RNA was reversely transcribed into cDNA via cDNA reverse transcription kit (Applied Biosystems; Thermo Fisher Scientific, Inc.) with the manufacturer’s protocol accordingly. MiR-22-3p was detected via mirVana™RT-qPCR miRNA assay kit (Catalog No. AM1558, Ambion; Thermo Fisher Scientific, Inc.) and standardized with U6 as an internal reference, and mRNA expression was examined via the SYBR Premixture Ex Taq™ kit (catalog number DRR041A, Takara Bio, Inc.) with glyceraldehyde-3-phosphate dehydrogenase (GAPDH). Gene expression was calculated using 2^−ΔΔCT^. The gene primer sequences were shown in Table 1.

**Table 1.** RT-qPCR primer sequence

	Primer sequence (5’ 3’)
GAPDH	Forward: 5’-ATGGGGAAGGTGAAGGTCG-3’
	Reverse: 5’-TTACTCCTTGGAGGCCATGTG-3’
U6	Forward: 5’-CTCGCTTCGGCAGCACATATACT-3’
	Reverse: 5’-ACGCTTCACGAATTTGCGTGTC-3’
MiR-22-3p	Forward: 5’- AAGCTGCCAGTTGAAGAACTGT-3’
	Reverse: 5’- CAGTGCGTGTCGTGGAGT-3’
TCF7L2	Forward: 5’- CTCCTCATCAATTGCACAGC’3’
	Reverse: 5’- GGAGCTGTGGGAATGTAACC −3’

### 2.9 Western blot

Total proteins were extracted from cells and tissues using Radio-Immunoprecipitation assay (RIPA) lysates (Thermo Fisher Scientific, Inc.). Bicinchoninic acid (BCA) reagent (Invitrogen; Thermo Fisher Scientific, Inc.) was implemented for determination of protein concentrations. The protein (30 µg) was isolated by 10% sulfate polyacrylamide gel electropheresis (SDS-PAGE), electroblotted onto Polyvinylidene fluoride (PVDF) membrane, which were then blocked with 5% skim milk in Tris-buffered saline with Tween 20 (TBST) (0.1% Tween-20; Sangon Biotech Co., Ltd.), and incubated with the following primary antibodies: GAPDH (ab8245, Abcam), TCF7L2 (13,838-1-AP, Proteintech), Wnt (ab15251, Abcam), β-catenin (ab32572, Abcam) and the membrane- horseradish peroxidase (HRP)-bound secondary antibody (ab6721, Abcam). The protein bands were observed via the enhanced chemiluminescence kit (Pierce; Thermo Fisher Scientific, Inc.). Protein expression was quantified with GAPDH as a sample control and Imagej software (version 4.0; Bio-Rad Laboratories, Inc.).

### 2.10 Xenografted tumors in nude mice

Twelve five-week-old nude mice were achieved from Hunan SJA Experimental Animal Co., Ltd and kept under laboratory conditions. All animal experiments and programs have been reviewed and approved by the Xiangya Hospital of Central South University

Animal Care and Use Committee. After adaptive feeding for one week, HOS cells (2 × 10^6^ cells) statically expressing mimic-NC and miR-22-3p-mimic were seeded into nude mice by subcutaneous injection (n = 6). The long and short diameters of tumors in mice were recorded weekly with a vernier caliper. Volume =  (long diameter × short diameter)2 × 0.5 [24]. Four weeks later, the mice were anesthetized with pentobarbital sodium (30 mg/kg) and the tumors were weighed. The tumor tissues were preserved in 4% paraformaldehyde.

### 2.11 Immunohistochemistry

The fresh tumor sample was embedded in paraffin and cut into 4 μm sections. The sections were incubated with anti-TCF7L2 (13,838-1-AP, Proteintech), Wnt (ab15251, Abcam), β-catenin (ab32572, Abcam), counterstained with hematoxylin (Sigma-Aldrich), and imaged via a microscope (Leica Microsystems), Mannheim, Germany).

### 2.12 Dual luciferase report experiment

The dual luciferase reporting assay was implemented as described afore [25]. The wild-type reporter gene (TCF7L2-WT) and mutant reporter gene (TCF7L2-MUT) of the TCF7L2 3ʹuntranslated regions (UTR) region containing miR-22-3p binding site were purchased from SynthGene Biotech (Nanjing, China). The sequence of WT-TCF7L2wasACAUACAAGGUGAAGGCAGCUG;The sequence of MUT-TCF7L2 was ACAUACAAGGUGAACCGUCGAG.TCF7L2-WT or -MUT was co-transfected with miR-22-3p-mimic or mimic-NC into MG63 and HOS cells using Lipofectamine 3000 (Invitrogen) on the grounds of the kit method. Then the relative luciferase activity of the cells was measured via a luciferase assay system (Promega, Shanghai, China).

### 2.13 Data analysis

The experimental results were expressed as mean ± standard deviation. SPSS 22 software was applied for data analysis, including Student’s t test and one-way analysis of variance (ANOVA), Tukey’s test for multiple variance corrections on the samples. The difference between the experimental groups was considered significant when *P* < 0.05.

## 3 Results

### 3.1 MiR-22-3p is of reduction in OS

**Figure 1.**
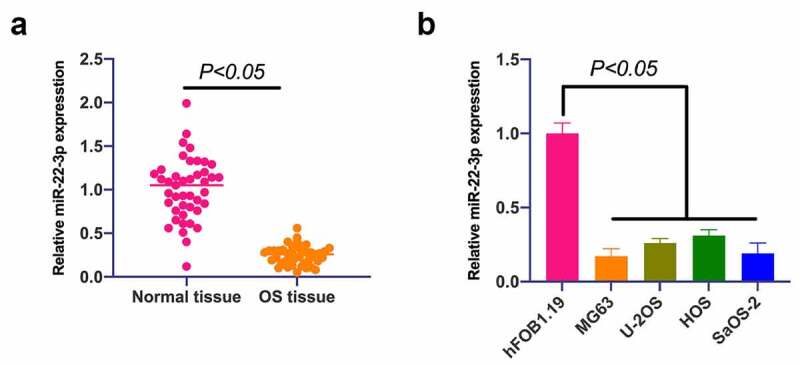
**MiR-22-3p is declined in OS, and up-regulation of miR-22-3p represses OS growth**. A. RT-qPCR to detect miR-22-3p expression in OS tissues and adjacent normal tissues; B. RT-qPCR to detect miR-22-3p expression in human normal osteoblast cell line hFOB1.19 and OS cell line MG63, U-2OS, HOS and SAOS-2. The values were shown as mean ± SD (Figure 1b, n = 3). The significance of each group was calculated using one-way ANOVA, and the variance correction via Tukey’s test.

MiR-22-3p in OS was firstly examined, exposing that miR-22-3p in OS was apparently reduced (Figure 1(a, b)), and implicated with distant metastasis of OS and tumor size (Table 2).

**Table 2.** MiR-22-3p is correlated with distant metastasis and tumor size of OS

Clinical pathologic parameters	Group	Cases (n = 45)	MiR-22-3p expression		*P*
			High expression group (n = 21)	Low expression group (n = 22)	
Age (years)	< 20	27	11	16	0.1677
	20 or more	16	10	6	
Gender	Male	32	13	19	0.0661
	Female	11	8	3	
Tumor size	< 8	20	15	5	0.0014
	8 or more	23	6	17	
Distant metastasis	Yes	29	11	18	0.0395
	No	14	10	4	
Entering stage	I + II	17	9	8	0.6633
	III	26	12	14	
Differentiation state	High	18	11	7	0.1719
	Low	25	10	15

### 3.2 Overexpression of miR-22-3p curbs OS growth and the Wnt/β-catenin pathway

**Figure 2.**
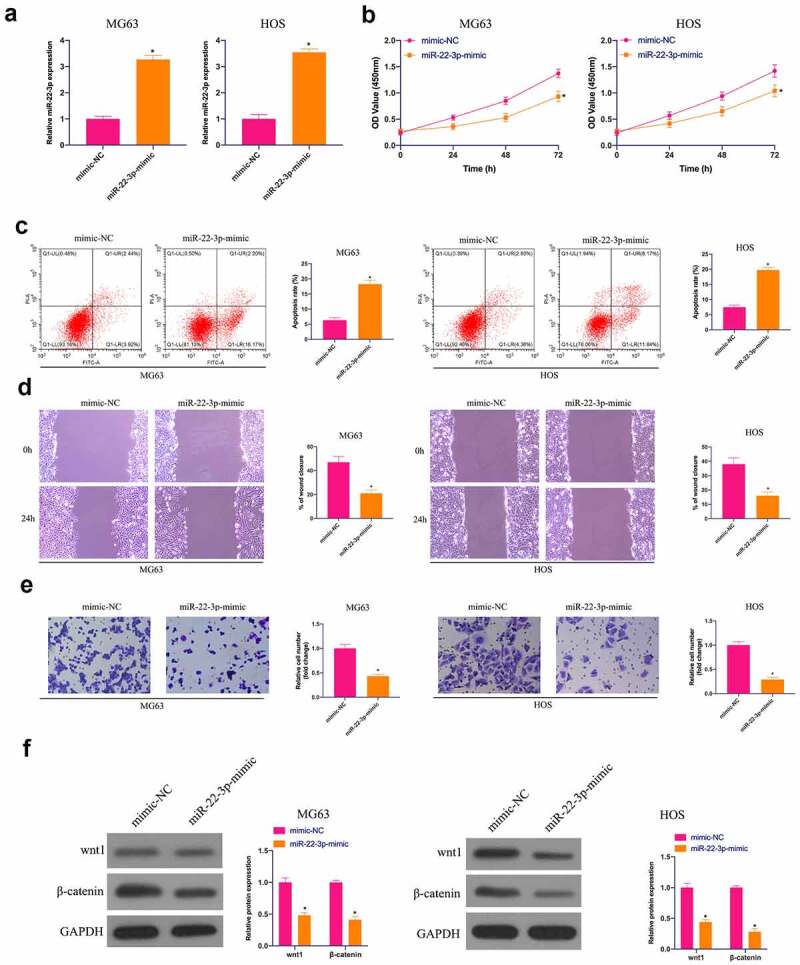
Overexpressed miR-22-3p prohibits OS growth and Wnt/β-catenin pathway. A. RT-qPCR to detect miR-22-3p in MG63 and HOS cells after transfection with miR-22-3p mimics; B. CCK-8 to detect MG63 and HOS cell proliferation after transfection with miR-22-3p mimics; C. Flow cytometry to detect the apoptosis rate of MG63 and HOS cells transfected with miR-22-3p mimics; D. Cell scratch for detection of MG63 and HOS cell migration after transfection with miR-22-3p mimics; E. Transwell detection of MG63 and HOS cell invasion after transfection of miR-22-3p mimics; F. Western blot for detection of Wnt and β-catenin expression in MG63 and HOS cells transfected with miR-22-3p mimics. The values were shown as mean ± SD (n = 3). The significance of each group was calculated using one-way ANOVA, and the variance correction via Tukey’s test. Vs. the mimic-NC group, * *P* < 0.05.

Next, the biological function of miR-22-3p was analyzed in OS. HOS cells with the highest miR-22-3p expression and MG63 cells with the lowest miR-22-3p were selected for functional verification experiment. First, MiR-22-3p in MG63 and HOS cells was upregulated (Figure 2(a)). There was a great deal of studies clarifying that up-regulated miR-22-3p constrains MG63 and HOS cell progression (Figure 2(b-e)). A large number of studies have been published that abnormal activation of the Wnt/β-catenin pathway can promote malignant behavior of cancer [26]. Therefore, the influence of elevated miR-22p-3p on Wnt/β-catenin pathway in MG63 and HOS cells was examined by Western blot, letting out that Wnt and β-catenin proteins in MG63 and HOS cells were prohibited via overexpression of miR-22-3p (Figure 2(f)), further suggesting that elevated miR-22-3p constrains OS growth and Wnt/β catenin pathway.

### 3.3 TCF7L2 is excessively expressed in OS and targeted by miR-22-3p

**Figure 3.**
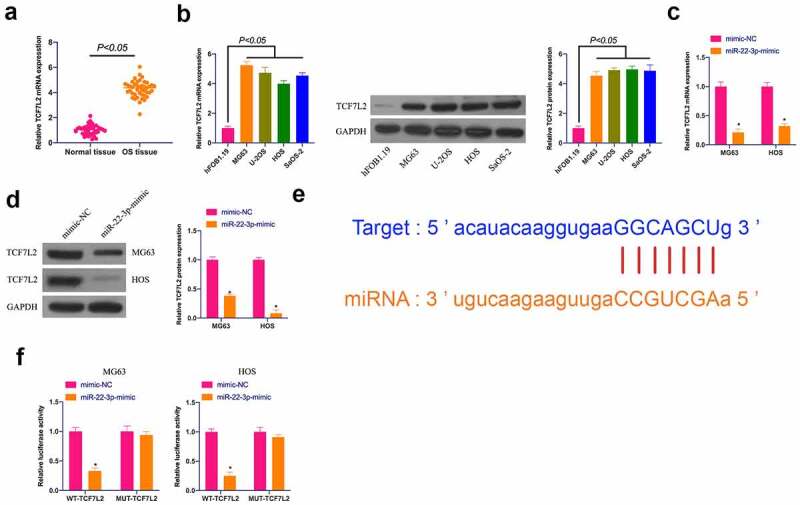
**TCF7L2 is highly expressed in OS and targeted by miR-22-3p**. A. TCF7L2 expression in OS tissues and adjacent normal tissues detected by RT-qPCR; B. TCF7L2 in OS cell lines MG63, U-2OS, HOS and SAOS-2 detected by RT-qPCR and Western blot; C, D. TCF7L2 in MG63 and HOS cells after transfection of miR-22-3p mimics detected by RT-qPCR and Western blot; E. MiR-22-3p and TCF7L2 potential binding sites queried through bioinformatics website http://starbase.sysu.edu.cn/; F. Targeting relationship between miR-22-3p and TCF7L2 detected by dual luciferase assay; The values were shown as mean ± SD (n = 3). The significance of each group was calculated using one-way ANOVA, and the variance correction via Tukey’s test. Vs. the mimic-NC group, * *P* < 0.05.

Next, the downstream target gene of miR-22-3p was researched. TCF7L2 is a familiar oncogene overexpressed in breast cancer, nephroblastoma, and gastric cancer [27–29], but its role in OS remains unsure. Therefore, TCF7L2 expression was first examined, clarifying that (Figure 3(a, b)) TCF7L2 in OS tissues and cells was clearly strengthened in contrast with adjacent normal tissues and cells, whereas suppressed via elevated miR-22-3p in MG63 and HOS cells (Figure 3(c, d)). Based on this, it was speculated that TCF7L2 might be the target gene of miR-22-3p. Through bioinformatics website http://starbase.sysu.edu.cn/ it was forecast the targeted binding sites between miR-22-3p and TCF7L2 (Figure 3(e)). The dual luciferase reporter experiment clarified (Figure 3(f)) that co-transfection of WT-TCF7L2 and miR-22-3p-mimic would reduce the luciferase activity, while that of MUY-TCF7L2 and miR-22-3p-mimic had no influences on the luciferase activity. These results suggest that TCF7L2 is the downstream target gene of miR-22-3p.

### 3.4 Upregulation of TCF7L2 promotes OS malignant behavior and activates Wnt/β catenin pathway

**Figure 4.**
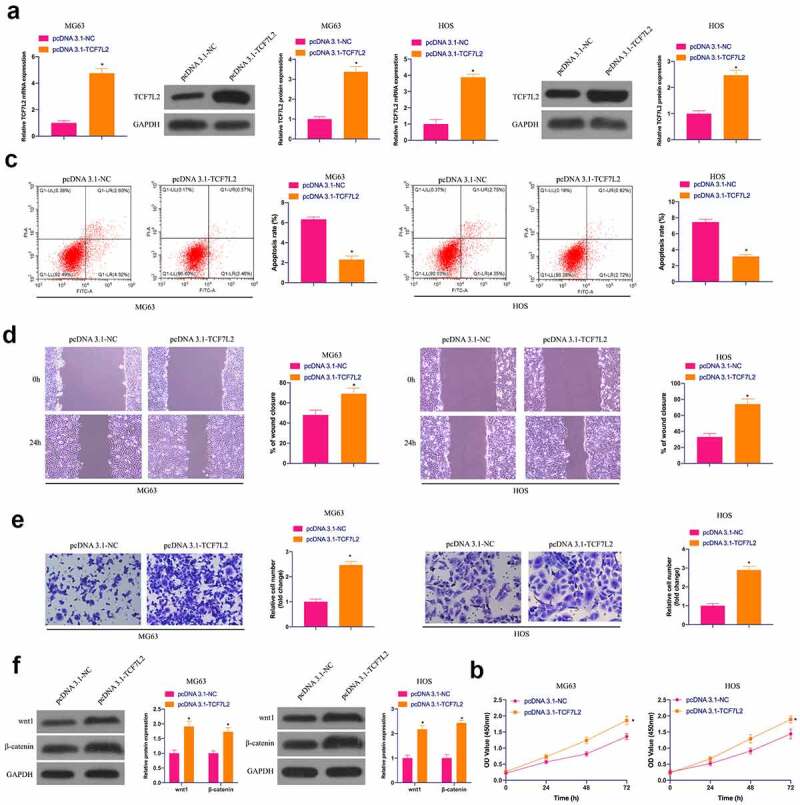
**Elevated TCF7L2 strengthens OS malignancy and results in activation of the Wnt/βcatenin pathway**. A. RT-qPCR and Western blot were applied to detect TCF7L2 in MG63 and HOS cells after transfection with pcDNA 3.1-TCF7L2; B. CCK-8 to detect MG63 and HOS cell proliferation after transfection with pcDNA 3.1-TCF7L2; C. Flow cytometry to detect the apoptosis rate of MG63 and HOS cells transfected with pcDNA 3.1-TCF7L2; D. Cell scratch for detection of MG63 and HOS cell migration after transfection with pcDNA 3.1-TCF7L2; E. Transwell for detection of MG63 and HOS cell invasion after transfection of pcDNA 3.1-TCF7L2; F. Western blot for detection of Wnt and β-catenin expression in MG63 and HOS cells transfected with pcDNA 3.1-TCF7L2. The values were shown as mean ± SD (n = 3). The significance of each group was calculated using one-way ANOVA, and the variance correction via Tukey’s test. Vs. the pcDNA 3.1-NC group, * *P* < 0.05.

The biological function of TCF7L2 was analyzed in OS. TCF7L2 was elevated via transfection of pcDNA 3.1-TCF7L2 in MG63 and HOS cells (Figure 4(a)). It was in a great many of studies proclaiming that the up-regulation of TCF7L2 facilitated the proliferation, migration and invasion, but prohibited the apoptosis rate of MG63 and HOS cells (Figure 4(b-e)). In the meantime, Wnt and β-catenin proteins were elevated via up-regulation of TCF7L2 (figure 4(f)). These results conclude that up-regulation of TCF7L2 can accelerate OS development and activate the Wnt/β-catenin pathway.

### 3.5 The accelerating effect of miR-22-3p knockdown on OS is reversed via simultaneously repressive TCF7L2

**Figure 5.**
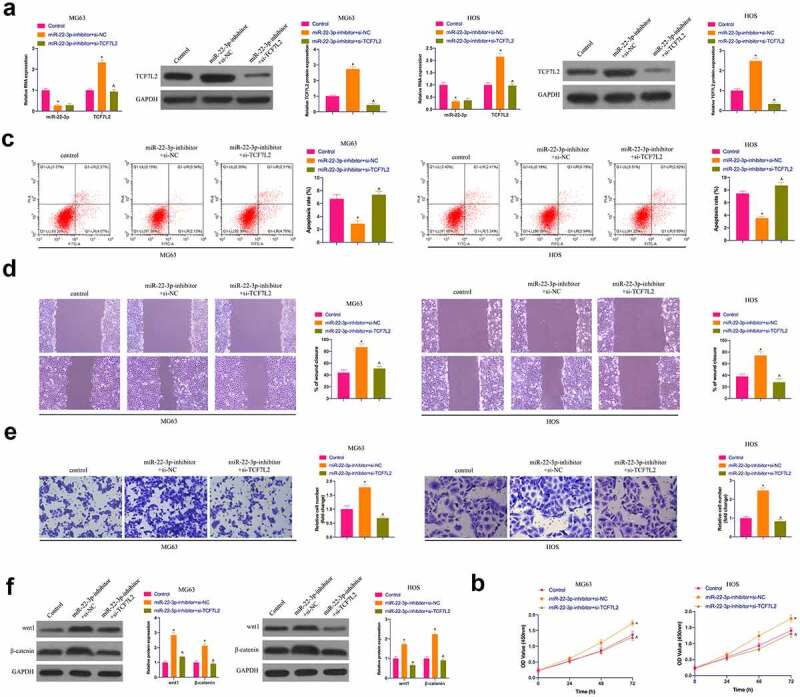
**The accelerating effect of knockdown miR-22-3p on OS is reversed via simultaneously repressive TCF7L2**. A. RT-qPCR and Western blot were applied to detect miR-22-3p and TCF7L2 in MG63 and HOS cells after transfection with miR-22-3p-inhibitor and si-TCF7L2; B. CCK-8 to detect MG63 and HOS cell proliferation after transfection with miR-22-3p inhibitor and si-TCF7L2; C. Flow cytometry to detect the apoptosis rate of MG63 and HOS cells transfected with miR-22-3p inhibitor and si-TCF7L2; D. Cell scratch for detection of MG63 and HOS cell migration after transfection with miR-22-3p inhibitor and si-TCF7L2; E. Transwell for detection of MG63 and HOS cell invasion after transfection of miR-22-3p inhibitor and si-TCF7L2; F. Western blot for detection of Wnt and β-catenin expression in MG63 and HOS cells transfected with miR-22-3p inhibitor and si-TCF7L2. The values were shown as mean ± SD (n = 3). The significance of each group was calculated using one-way ANOVA, and the variance correction via Tukey’s test. Vs. the Control group, * *P* < 0.05; Vs. the miR-22-3p-inhibitor + si-NC group, ^ *P* < 0.05.

For investigating whether miR-22-3p controls OS development by targeting TCF7L2, TCF7L2 was silenced while declining miR-22-3p. As manifested in Figure 5(a), TCF7L2 was strengthened via silencing of miR-22-3p. however, miR-22-3p was not influenced via depression of TCF7L2. What’s more, depressed miR-22-3p facilitated the proliferation, invasion and migration but inhibited apoptosis of MG63 and HOS cells, which was reversed by simultaneous silencing of TCF7L2 (Figure 5(b-e)). Western blot conveyed that Wnt and β-catenin proteins in MG63 and HOS cells were up-regulated via miR-22-3p silencing, but prohibited via TCF7L2 silencing (Figure 5(f)), declaring that miR-22-3p can repress the malignant behavior of OS by controlling TCF7L2.

### 3.6 The repressive effect of elevated miR-22-3p on OS is reversed via the simultaneous overexpression of TCF7L2

**Figure 6.**
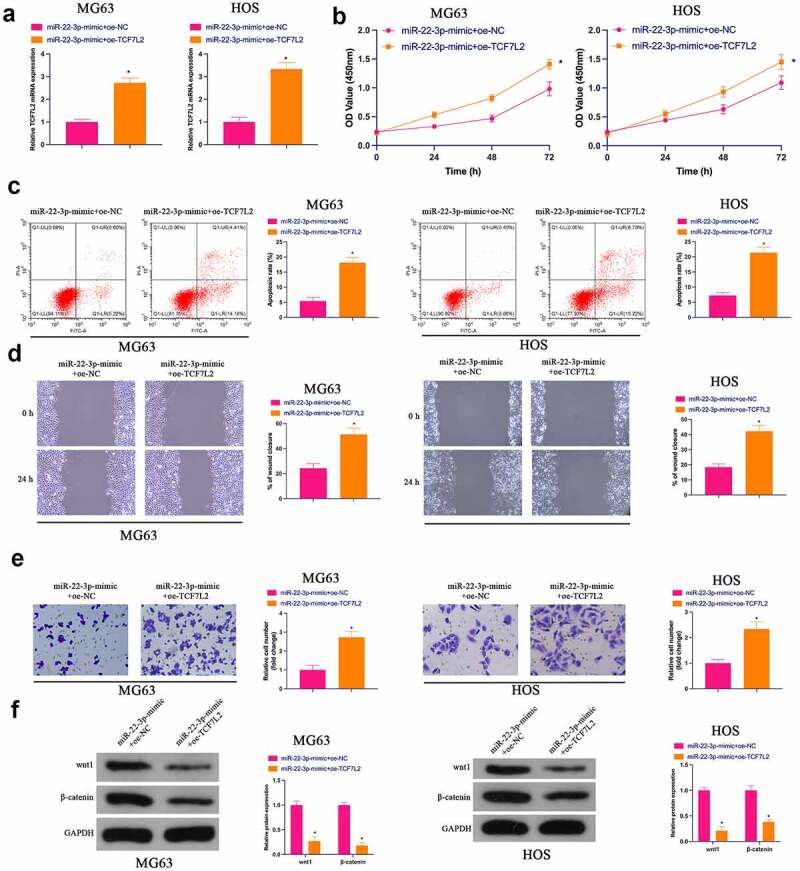
**Repression of OS via elevated miR-22-3p is reversed via simultaneous overexpression of TCF7L2**A. RT-qPCR and Western blot were applied to detect miR-22-3p and TCF7L2 in MG63 and HOS cells after transfection with miR-22-3p-mimic and pcDNA 3.1-TCF7L2; B. CCK-8 to detect MG63 and HOS cell proliferation after transfection with miR-22-3p-mimic and pcDNA 3.1-TCF7L2; C. Flow cytometry to detect the apoptosis rate of MG63 and HOS cells transfected with miR-22-3p-mimic and pcDNA 3.1-TCF7L2; D. Cell scratch for detection of MG63 and HOS cell migration after transfection with miR-22-3p-mimic and pcDNA 3.1-TCF7L2; E. Transwell for detection of MG63 and HOS cell invasion after transfection of miR-22-3p-mimic and pcDNA 3.1-TCF7L2; F. Western blot for detection of Wnt and β-catenin expression in MG63 and HOS cells transfected with miR-22-3p-mimic and pcDNA 3.1-TCF7L2. The values were shown as mean ± SD (n = 3). The significance of each group was calculated using one-way ANOVA, and the variance correction via Tukey’s test. Vs. the miR-22-3p-mimic+pcDNA 3.1 group, * *P* < 0.05.

Subsequently, miR-22-3p-mimic and oe-TCF7L2 were co-transfected into MG63 and HOS cells. As clarified in Figure 6(a), the repressive effect of elevated miR-22-3p on TCF7L2 expression was prevented via simultaneous overexpression of TCF7L2.Functional experiments manifested that the repressive effect of elevated miR-22-3p on OS proliferation, invasion and migration and the facilitation of apoptosis were reversed via overexpression of TCF7L2 (Figures 6(b-f)). Shortly, MiR-22-3p repressed OS malignant activities via modulating TCF7L2.

### 3.7 *MiR-22-3p restrains OS tumor growth* in vivo

**Figure 7.**
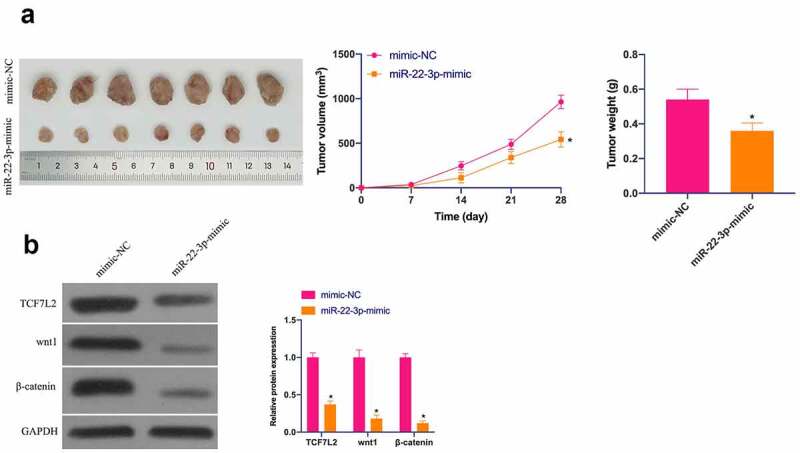
MiR-22-3p depresses OS *in vivo*. A. Representative pictures of the tumor; B. Tumor volume; C. Tumor weight; D. TCF7L2, Wnt and β-catenin changes in the tumor after elevated miR-22-3p detected by IHC. The values were shown as mean ± SD (n = 6). The significance of each group was calculated using one-way ANOVA, and the variance correction via Tukey’s test. Vs. the mimic-NC group, * *P* < 0.05.

Finally, the influence of miR-22-3p/TCF7L2 axis on OS tumor growth was further examined in a xenograft assay in nude mice. As suggested in Figure 7(a-c), elevated miR-22-3p repressed tumor volume and weight. In addition, TCF7L2, Wnt and β-catenin in tumors were prohibited via elevated miR-22-3p (Figure 7(d)). Briefly, miR-22-3p represses OS growth by regulating TCF7L2 *in vivo*.

## 4 Discussion

MiRNA, a type of abundant and relatively stable non-coding RNA in tissues and blood, is once regarded as the metabolic by-products of biological processes [30]. However, recently, increasing studies have clarified the emphasis of miRNA in various diseases, especially in cancers [31,32]. MiRNAs are able to influence the proliferation, apoptosis, metastasis and drug resistance of cancer by binding with downstream proteins [33]. In this study, it was affirmed that miR-22-3p depressed OS progression via knockout of TCF7L2. Furthermore, miR-22-3p prohibited the Wnt/β-catenin pathway in OS cells by targeting TCF7L2.

Malignant proliferation of cancer cells will consume plenty of nutrients and energy in the body and cause the failure of other organs in the body. Previous studies have manifested that up-regulated miR-22-3p constrains malignant proliferation in gastric cancer, non-small cell lung cancer, and acute lymphoblastic leukemia [34–36]. It was confessed in this study that the up-regulated miR-22-3p depressed the proliferation ability and tumor growth of OS cells, which further revealed the tumor suppressive influence of miR-22-3p. A recent study has published that the number of invaded and migrated cells in colorectal cancer is declined via upregulation of miR-22-3p [37]. Enhanced invasion and migration can facilitate the spread of cancer cells and cause serious damage to normal tissues. In this study, up-regulated miR-22-3p constrained the invasion and migration of OS, suggesting that miR-22-3p is a crucial regulator of distal metastasis and malignant invasion of cancer. Notably, a recent research has manifested that miR-22-3p is related to the regulation of chemical resistance of bladder cancer [38]. Tumor chemical resistance is an key resistance to cancer treatment at present. It is speculated that miR-22-3p also plays a similar role in OS, which needs to be explored in subsequent studies. MiRNAs are frequently controlled by circular RNAs (circRNAs) or long non-coding RNAs (lncRNAs), which can act as sponges of miRNAs to adsorb miRNA expression in the body [39]. MiR-22-3p is controlled by circRNAs or lncRNAs in all kinds of diseases. For example, lncRNA transcripts of myocardial infarction (lncMIAT) can impact diabetic cardiomyopathy by constraining miR-22-3p for the up-regulation of Death-associated protein kinase 2 (DAPK2) [40]. CircRNA mitogen-activated protein kinase kinase kinase 5 (CircMAP3K5), as a sponge for miR-22-3p, facilitates intimal hyperplasia throughTen-Eleven-Translocation (TET)2-mediated smooth muscle cell differentiation [41]. CircRNA Enolase 1 (ENO1) regulating the miR-22-3p/eNO1 axis accelerates glycolysis and tumor progression of lung adenocarcinoma [42]. Therefore, there may be a variety of competing endogenous RNA (ceRNA)s controlling miR-22-3p in OS to affect the biological progress, and the upstream regulatory molecules of miR-22-3p need to be further explored.

TCF7L2 is a nuclear transcription factor found to be abnormally expressed in liver, breast and colorectal cancers [43–45]. In this study, it was manifested that TCF7L2 was the downstream target gene of miR-22-3p. The up-regulation of TCF7L2 facilitated OS development, explaining a proto-oncogene role of TCF7L2 in OS. There is a great deal of studies revealing that TCF7L2 facilitates disease development with interaction of Wnt/β-catenin pathway. These include brain development, mental disorders, colorectal cancer, breast cancer, weight gain and insulin resistance [44–47]. TCF transcription factor family is the main nuclear mediator of Wnt/β-catenin signaling pathway [48] TCF7 and TCF7L2 can perform as both activators and repressors of Wnt targets, depending on the context [49]. It was discovered in the study that miR-22-3p prevented the Wnt/β-catenin pathway in OS cells via targeted regulation of TCF7L2. It was speculated that TCF7L2 is cancer-promoting in OS with controlling the Wnt/β-catenin pathway. However, it is not clear whether Wnt/β-catenin is the only target of TCF7L2, which requires to be further examined in subsequent Wnt/β-catenin knockout models. It is advised that miR-22-3p/TCF7L2 serves as a potential target for OS therapy, but the other key target genes of miR-22-3p is not supposed to be ruled out. What’s more, Wnt/β-catenin also participates in cancer drug resistance and EMT [50]. Therefore, miR-22-3p/TCF7L2 may be crucial in OS resistance and EMT as well, whereas whose specific mechanism needs to be further explored [51].

## 5 Conclusion

In short, it is suggested that miR-22-3p restrains OS cell progression and prevents Wnt/β-catenin pathway activation via suppression of TCF7L2. In the future, miR-22-3p/TCF7L2 axis can be applied as a hopeful target for OS treatment.
